# Influence of antiresorptive/antiangiogenic therapy on changes in periodontal and oral tissue structures: a histomorphometrical analysis in rats

**DOI:** 10.1007/s00784-023-05359-7

**Published:** 2023-11-01

**Authors:** Ausra Ramanauskaite, Katharina Mangold, Ninad Padhye, Karina Obreja, Fanya Borschert, Iulia Dahmer, Frank Schwarz

**Affiliations:** Department of Oral Surgery and Implantology, Goethe University, Carolinum, Theodor-Stern-Kai 7; Building 29, 60596 Frankfurt am Main, Germany

**Keywords:** Animal experiment, Antiresorptive therapy, Antiangiogenic therapy, Periodontal space, Histological technique, Treatment

## Abstract

**Objective:**

The objective of this study was to investigate the influence of various antiresorptive and antiangiogenic medications on morphological changes in periodontal and oral tissue structures.

**Materials and methods:**

Fifty-five Wistar rats randomly received dual application (i.e., at baseline and after 12-weeks) one of the following medications: (1) amino-bisphosphonate [zoledronate (Zo)], (2) RANKL inhibitor [denosumab (De)], (3) antiangiogenic [bevacizumab (Be)], (4) Zo + Be, (5) De + Be or (6) no medication [Control (Co)]. Periodontal and oral tissue biopsies were obtained at 17 (*n* = 21 animals, Phase 1, (De = 3, De + Be = 3, Zo = 5, Be = 3, Zo + Be = 2, Co = 5) and 29 (*n* = 34 animals, (De = 8, De + Be = 6, Zo = 2, Be = 7, Zo + Be = 4, Co = 7, Phase 2) weeks after the second drug application. The following outcomes were histomorphometrically assessed: periodontal space width in the coronal (PLS-C, mm) and apical sections (PLS- A), number of empty alveolar bone lacunae in the coronal, apical sections and at the apex at respective tooth sites (EL – C, EL- A, EL- Ap), mucosal thickness at edentulous alveolar ridge areas (MT, mm), and, when present, associated areas of inflammatory cell infiltrates (ICI, mm^2^).

**Results:**

Comparable mean PLS-C, PLS-A, ET-A, ET-C, ET-Ap, and MT values were observed in all experimental groups after Phases 1 and 2. The presence of ICI was identified in 3 animals in the Co group (Phase 1: 1, Phase 2: 2), and 17 animals in the test groups (Phase 1: 4; Phase 2: 14). The estimated ICI surface area was significantly higher in the Zo + Be group, followed by the Zo and Be groups compared to that measured in the Co group. The time (i.e., Phases 1 and 2) was not found to be a predictor for the extent of the ICI area. In all groups, the EL-C, EL-A, and EL-Ap values were significantly higher after Phase 2 compared to those assessed after Phase 1. The MT values were significantly reduced in all groups after Phase 2 compared to those measured after Phase 1.

**Conclusions:**

The present evaluation was not able to find any morphological effects of different antiresorptive and antiangiogenic medications on periodontal and oral tissue structures. The presence of inflammatory cell infiltrates was more frequently observed in the animals administered with antiresorptive and antiangiogenic medications as well as combinations thereof.

**Clinical relevance:**

Administration of antiresorptive and antiangiogenic medications may be capable of inducing inflammatory reactions in periodontal tissues.

## Introduction

Medication-related osteonecrosis of the jaw (MRONJ) is one of the possible side effects of the administration of antiresorptive or antiangiogenic drugs [1–3]. As documented in numerous preclinical and clinical investigations, antiresorptive medications, including bisphosphonates (e.g., zoledronic acid), inhibitors for the receptor activator of nuclear factor-κB ligand (RANKL; e.g., Denosumab), and antiangiogenic therapy (e.g., Bevacizumab, a vascular endothelial growth factor inhibitor), decrease bone turnover, increase bone density, inhibit angiogenesis, and subsequently decrease bone vascularity [4–11]. As such, radiographically detected thickening of the lamina dura, alveolar crest or sclerosis of the alveolar margin, and widening of the periodontal ligament space (PLS) are frequent findings in patients receiving antiresorptive therapy [12]. In fact, an increased width of the PLS could be detected in 83% of the patients diagnosed with MRONJ following tooth extraction, thus suggesting that changes in periodontal tissues may potentially predispose extraction sites to MRONJ [13].

In line with the radiographic findings noted in humans, one recent volumetric analysis of the periodontal and bone microstructure at noninfected teeth in rabbits repeatedly administrated with zoledronic acid (test group) also showed a marked increase in PLS, which was associated with a trend toward an increase in the adjacent trabecular bone thickness compared to the negative control group [14]. Similar outcomes were obtained in previous experimental studies employing rat models depicting an apparent correlation between the application of zoledronic acid and an increase in bone density, trabeculae thickness, and decreased alveolar bone vascularization at healthy teeth sites [15, 16]. On the other hand, at experimentally induced peri-implantitis lesions in rats, no marked effects of different antiresorptive/antiangiogenic medications were noted on either the extent of the inflammatory lesion or the surrounding bone microstructure [17].

Considering the crucial effect of antiresorptive/antiangiogenic therapy on the pathogenesis of MRONJ, a better understanding of the effect of the aforementioned medications on structural changes of various oral tissues is required [4, 5, 7]. Currently, there is a lack of data on the potential effect of various antiresorptive/antiangiogenic medications and their combinations on morphological changes of periodontal and/or oral tissues. Therefore, the present study aimed to investigate the potential effect of antiresorptive/antiangiogenic medications administered to healthy animals on morphological changes in periodontal and oral tissues. It is hypothesized that the application of antiresorptive/antiangiogenic medications alter the structure of PLS, surrounding alveolar bone and mucosal thickness.

## Materials and methods

### Study design and animals

This study is a secondary analysis reporting on the histological outcomes of tissue samples obtained from two previous experimental studies investigating the influence of the administration of antiresorptive/antiangiogenic therapy on the histological characteristics of experimentally induced peri-implantitis lesions [17] and the outcomes of peri-implantitis treatment [18]. Fifty-five 6-month-old albino rats of the Wistar strain (mean weight: 0.586 ± 0.53 g; Janvier Labs, Sulzfeld, Germany) were available for this analysis. Throughout the experiments, the animals were housed in appropriately dimensioned cages under standard conditions of temperature, in a light-controlled environment, and were provided water and a special diet ad libitum. The study protocol considered the 3Rs (replace, reduce, and refine) guidelines for animal experimentation and was approved by the appropriate local authority (Regierungspräsidium Darmstadt, Germany). The current writing followed the ARRIVE Guidelines 2.0 [19].

### Study design

**Fig. 1:**
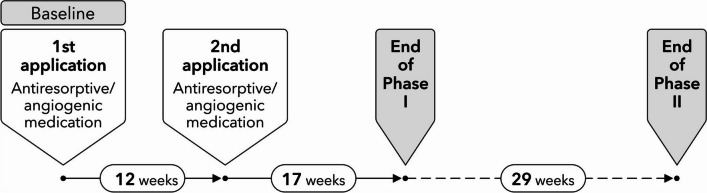
Illustration depicting the experimental phases of the study (Phase 1 and Phase 2)

All animals randomly received dual applications of the following medications at baseline and after 12 weeks: (1) amino-bisphosphonate (Zoledronate 5 mg/kg intravenous, Ribometa® 4 mg/5 ml, Hikma Pharma, Gräfelfing, Germany) (Zo); (2) RANKL inhibitor (Denosumab 60 mg/kg subcutaneous, Prolia®, Amgen, Munich, Germany) (De); (3) antiangiogenic medication (Bevacizumab 5 mg/kg intravenous, Avastin® 400 mg/16 ml, Roche Pharma, Grenzach-Wyhlen, Germany) (Be); (4) Zo + Be; (5) De + Be; or (6) no medication serving as the control group (Co). Twenty-one animals were sacrificed after 17 weeks following the second application of the medications (Phase 1), and 34 animals were sacrificed after 29 weeks of the second application of the medications (Phase 2) (Fig. 1).

### Histological processing

Euthanasia was performed with an overdose of pentobarbitone (100 mg/kg). The jaw specimens (Phase 1: upper and lower jaws; Phase 2: upper jaws) were fixed in a 10% neutral buffered formalin solution for 10 days. Then, the specimens were decalcified using 10% Ethylenediaminetetraacetic acid (EDTA), under agitation for 12 weeks, and processed for conventional paraffin embedding. For sections obtaining, each block was aligned parallel to the occlusal plane of the 1st/2nd molar and cut in a bucco-oral direction, with the microtome set at 3–4 μm. Finally, the serial most central serial sections were selected and manually stained with hematoxylin and eosin, following a standardized protocol. For the present analysis, histological samples of alveolar process containing the 2nd molar in the upper jaw (Phase 1 and Phase 2), and 1st molar in the lower jaw (Phase 1) were analyzed.

### Histological and histomorphometrical analysis

**Fig. 2:**
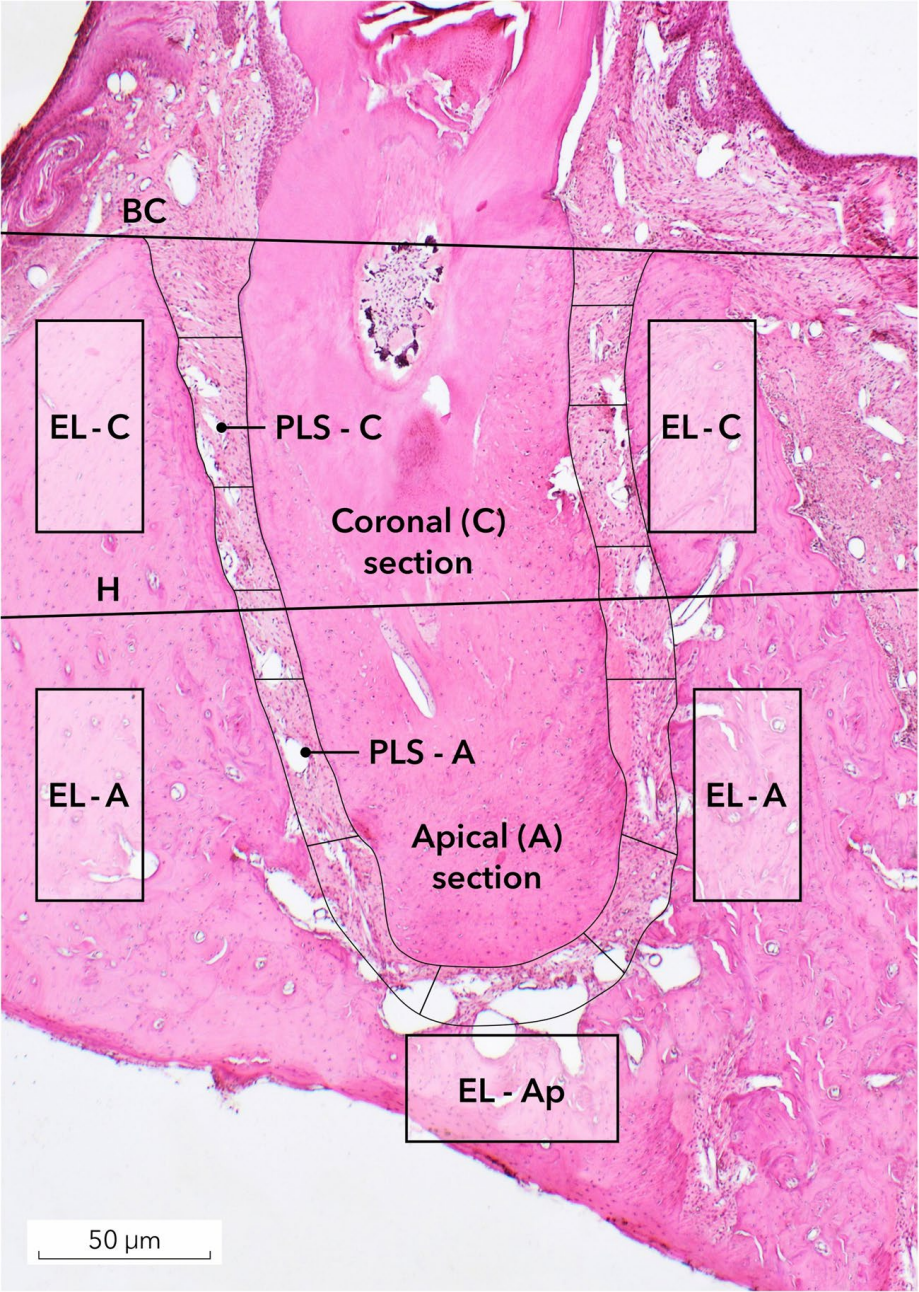
Landmarks and outcomes defined for the histomorphometrical analysis: H – horizontal line dividing root in coronal and apical sections; BC – line connecting bone crest vestibular and lingual/palatinal; PLS – periodontal ligament space; C – coronal section; A – apical section; Ap – apex area (De group, Phase 1)

Digital images were obtained from each specimen and evaluated using a specialized software (cellSens, Olympus). The following landmarks were identified in the histological sections at each tooth site (Fig. 2):

- The line connecting the bone crest at the oral and vestibular aspects (BC);
- The horizonal line (H) dividing the tooth root into the coronal (C) and apical (A) sections;
- The PLS marking a space between the tooth and surrounding alveolar bone;
- Rectangles of 0.6 mm^2^ depicting the alveolar bone surrounding the tooth root in sections C and A and the apex (Ap).

**Fig. 3:**
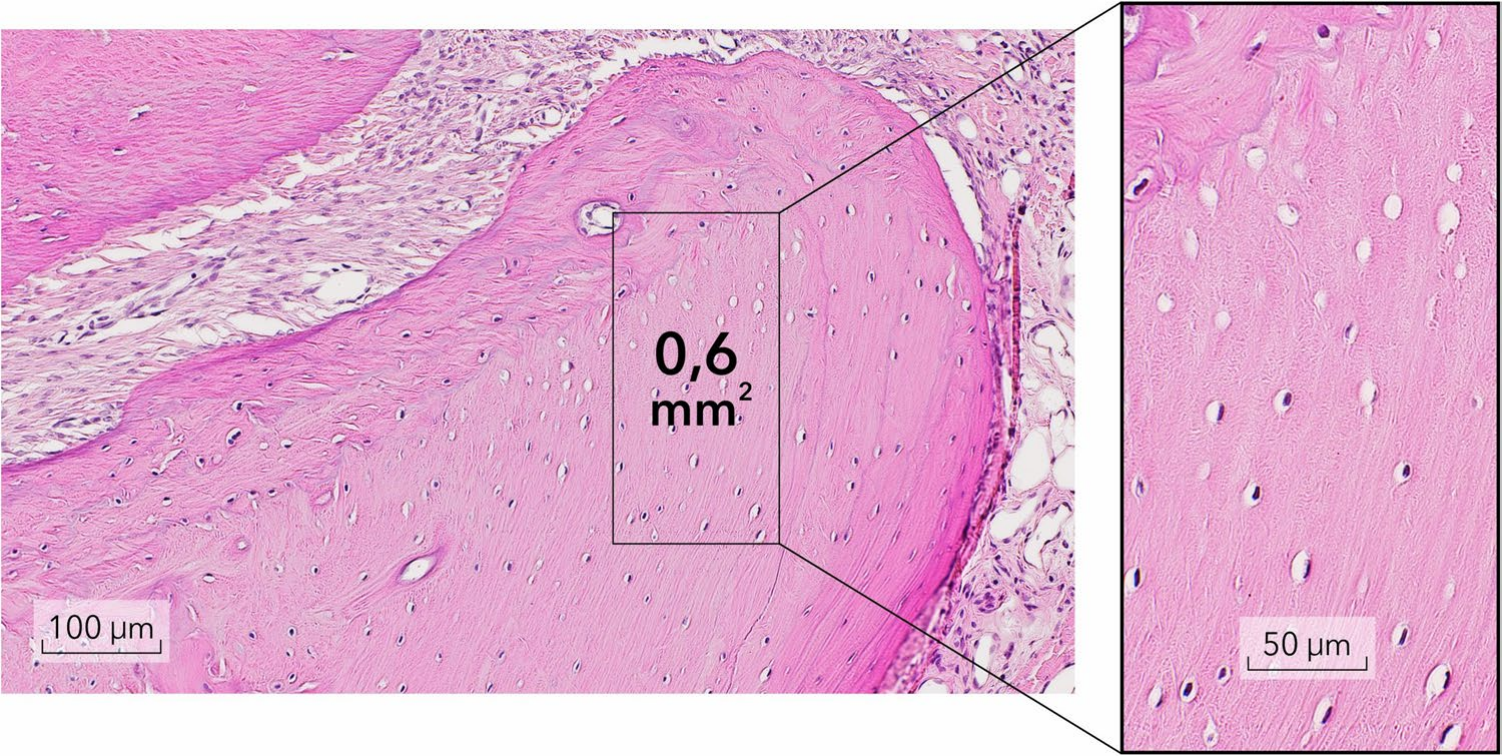
The number of empty lacunae (MT) were measured in the selected rectangles (De + Be group, Phase 2)

**Fig. 4:**
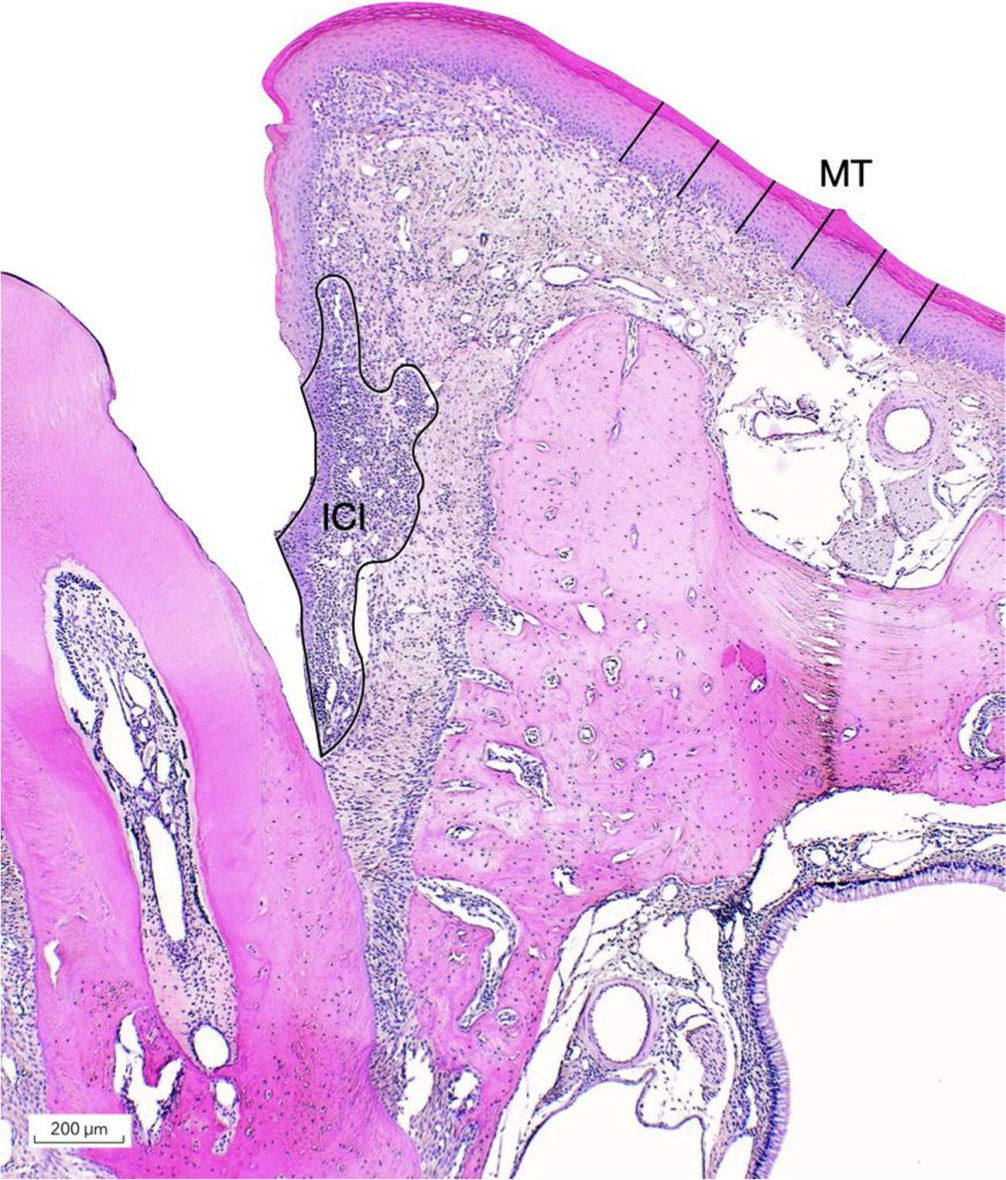
The epithelial thickness (MT) was measured at six points, and when present, the surface area of inflammatory cell infiltrate (ICT) was (Be group, Phase 2)

Accordingly, the following measurements were assessed:

1. PLS width in section C (PLS-C; mm);
2. PLS width in section A (PLS-A; mm);
3. Number of empty lacunae in the selected rectangles in section C (EL-C; Fig. 3);
4. Number of empty lacunae in the selected rectangles in section A (EL-A);
5. Number of empty lacunae in the selected Ap section (EL-Ap);
6. The mucosal thickness at edentulous alveolar ridge areas, measured from the outer keratinized layer to the basal membrane (MT; mm; Fig. 4);
7. When present, the surface area (mm^2^) of the inflammatory cell infiltrate (ICI) was measured in the connective tissues surrounding the tooth.

One previously calibrated examiner (K. M.) performed all measurements. Calibration was accepted when repeated measurements of *n* = 5 sections were similar at the > 95% level.

### Statistical analysis

The statistical analysis was performed using commercially available software (SPSS, 19.0, Chicago, IL, USA, R and Rstudio). The mean values, standard deviations, and confidence intervals for each variable (95% CI) were calculated, considering animals as a statistical unit. For the PLS and EL assessments in sections C and A, the mean values of the measurements assessed at the oral and lingual/palatinal aspects were used. For the MT and PLS assessments, mean values of the six measurements were computed. At multirooted teeth, the mean values assessed at both roots were obtained.

Linear regression analyses were conducted to assess the influence of the experimental group (Co, Zo, De, Be, Zo + Be, De + Be) and of time (i.e., Phase 1 and Phase 2) as predictors for the investigated outcome measures (i.e., PLS-C, PLS-A, EL-C, EL-A, EL-Ap, ICI, and MT). A Shapiro–Wilk test with a significance level of 5% was used to assess the normality of the data. When data were not normally distributed, logarithmic transformation was employed (i.e., for PLS-C, EL-C, ICI, and MT). Differences in the measurements between upper and lower jaws in the first phase were investigated using Wilcoxon-Mann–Whitney-U tests. Results were found to be significant at *p* < 0.05.. The effect sizes (Cohen’s d) for the comparisons of the control and test groups were estimated based on the means and standard deviations of the surface area (mm^2^) of the ICI assessed in the peri-implant mucosa of control and test (i.e. Zo, De, Be, Zo + Be, De + Be) animals, as reported recently [17]. For the sample sizes in our study (control (*n* = 12), Zo (*n* = 7), De (*n* = 11), Be (*n* = 10), Zo + Be (*n* = 6), De + Be (*n* = 9)), type I error alpha = 5% and estimated effect sizes (Zo (d = 0.93), De (d = 0.66), Be (d = 0.61), Zo + Be (d = 0.97), De + Be (d = 1.18)), the post hoc-power analysis led to a power of 47% (i.e. Zo), 34% (i.e. De), 28% (i.e. Be), 48% (i.e. Zo + Be), and 75% (i.e. De + Be) (linear regression, R: A language and environment for statistical computing, Vienna, Austria), respectively.

## Results

The mean PLS, EL, ICI, and MT values in various groups after Phases 1 and 2 are reported in Table 1.

**Table 1:** Histomorphometrical analyses (mean ± SD; 95% CI) of periodontal ligament space width (PLS, mm), number of empty lacunae (EL), inflammatory cell infiltrate (ICI, mm^2^), and mucosal thickness (MT, mm) in different groups after Phase 1 (*n* = 16 animals) and Phase 2 (*n* = 34 animals)

*Phase 1 (17 weeks; n* = *21 animals)*							
Group	PLS-C (mm)	PLS-A (mm)	EL-C (No.)	EL-A (No.)	EL-Ap (No.)	ICI (mm^2^)	MT (mm)
Zo (*n* = 5)						*n* = 1	
Mean SD 95% CI Min Max	0.17 0.1 (0.08, 0.26) 0.06 0.32	0.15 0.08 (0.09, 0.22) 0.06 0.27	5.9 2.33 (3.86, 7.94) 2.5 9	6.5 2.87 (3.98, 9.02) 2 9	2.3 3.19 (-0.5, 5.1) 0 6.5	0.9 - - 0.9 0.9	0.26 0.02 (0.24, 0.28) 0.23 0.29
De (*n* = 3)						*n* = 0	
Mean SD 95% CI Min Max	0.14 0.02 (0.12, 0.16) 0.13 0.16	0.14 0.02 (0.12, 0.16) 0.13 0.16	2.83 0.76 (1.97, 3.7) 2 3.5	2.08 1.38 (0.53, 3.64) 0.75 3.5	4 3.46 (0.08, 7.92) 0 6	-	0.26 0.07 (0.19, 0.34) 0.19 0.32
Be (*n* = 3)						*n* = 1	
Mean SD 95% CI Min Max	0.31 0.16 (0.13, 0.48) 0.18 0.48	0.24 0.18 (0.04, 0.44) 0.14 0.44	6.5 4.09 (1.87, 11.13) 3 11	2.67 2.08 (0.31, 5.02) 1 5	1 1 ( -0.13, 2.13) 0 2	0.1 - - 0.1 0.1	0.36 0.15 (0.19, 0.53) 0.27 0.54
Zo + Be (*n* = 2)						*n* = 2	
Mean SD 95% CI Min Max	0.15 0.02 (0.12, 0.17) 0.13 0.16	0.15 0.04 (0.09, 0.21) 0.12 0.18	5.25 1.77 (2.8, 7.7) 4 6.5	5.25 1.06 (3.78, 6.72) 4.5 6	6.75 0.35 (6.26, 7.24) 6.5 7	0.38 - - 0.38 0.38	0.26 0.06 (0.18, 0.35) 0.22 0.31
De + Be (*n* = 3)						*n* = 0	
Mean SD 95% CI Min Max	0.23 0.06 (0.16, 0.3) 0.16 0.26	0.19 0.03 (0.16, 0.23) 0.17 0.23	3.83 1.76 (1.85, 5.82) 2 5.5	5.67 2.08 (3.31, 8.02) 4 8	3.67 3.21 (0.03, 7.3) 0 6	-	0.22 0.06 (0.15, 0.28) 0.18 0.28
Control (*n* = 5)						*n* = 1	
Mean SD 95% CI Min Max	0.21 0.11 (0.11, 0.31) 0.09 0.37	0.15 0.04 (0.11, 0.19) 0.12 0.23	8.8 4.86 (4.54, 13.06) 4.5 16.5	4.1 2.38 (2.01, 6.19) 1.5 7.5	3.2 3.35 (0.27, 6.13) 1 9	0.1 - - 0.1 0.1	0.29 0.04 (0.25, 0.32) 0.22 0.33
*Phase 2 (29 weeks; n* = *34 animals)*							
Group	PLS-C (mm)	PLS-A (mm)	EL-C (No.)	EL-A (No.)	EL-Ap (No.)	IICI (mm^2^)	MT(mm)
Zo (*n* = 2)						*n* = 0	
Mean SD 95% CI Min Max	0.16 0.02 (0.12, 0.19) 0.14 0.17	0.12 0.03 (0.08, 0.17) 0.1 0.15	12.5 2.12 (9.56, 15.44) 11 14	9.75 4.6 (3.38, 16.12) 6.5 13	3.75 2.47 (0.32, 7.18) 2 5.5	-	0.17 0.07 (0.08, 0.27) 0.13 0.22
De (*n* = 8) Mean SD 95% CI Min Max	0.16 0.05 (0.13, 0.19) 0.12 0.27	0.19 0.06 (0.15, 0.23) 0.13 0.32	13.16 7.14 (8.21, 18.1) 3.5 27.5	10.34 3.87 (7.66, 13.02) 6 15.5	6.69 3.46 (4.29, 9.09) 1 11	*n* = 4 0.05 0.03 (0.03,0.07) 0.02 0.1	0.21 0.09 (0.15, 0.27) 0.11 0.41
Be (*n* = 7)						*n* = 5	
Mean SD 95% CI Min Max	0.18 0.05 (0.14, 0.22) 0.1 0.24	0.19 0.08 (0.13, 0.25) 0.09 0.33	9.75 7.94 (3.87, 15.63) 2.5 26	9.86 5.35 (5.9, 13.82) 3 17.25	4.86 2.93 (2.69, 7.02) 2 10.75	0.12 0.09 (0.06, 0.19) 0.03 0.26	0.22 0.09 (0.15, 0.28) 0.13 0.38
Zo + Be (*n* = 4)						*n* = 2	
Mean SD 95% CI Min Max	0.23 0.07 (0.16, 0.29) 0.17 0.31	0.21 0.05 (0.16, 0.26) 0.15 0.26	13.94 4.48 (9.55, 18.33) 10.75 20.25	9.12 5.65 (3.59, 14.66) 5.5 17.5	6.62 3.12 (3.57, 9.68) 2.5 9.5	1.1 0.36 (0.74, 1.45) 0.84 1.35	0.28 0.06 (0.22, 0.34) 0.22 0.33
De + Be (*n* = 6)						*n* = 3	
Mean SD 95% CI Min Max	0.16 0.11 (0.07, 0.24) 0.08 0.37	0.19 0.09 (0.12, 0.26) 0.13 0.36	9.92 5.34 (5.64, 14.19) 3 15.5	9.92 3.11 (7.43, 12.4) 7 15.5	4.27 4.02 (1.05, 7.49) 0.5 10	0.04 0.02 (0.02, 0.06) 0.03 0.07	0.22 0.04 (0.18, 0.25) 0.17 0.28
Control (*n* = 7)						*n* = 2	
Mean SD 95% CI Min Max	0.15 0.04 (0.12, 0.18) 0.11 0.21	0.14 0.03 (0.12, 0.16) 0.11 0.19	11.29 4.06 (8.28, 14.29) 4.5 17.5	10.82 5.53 (6.73, 14.92) 3.75 18.5	7.25 2.72 (5.24, 9.26) 3.25 12	0.02 0 (0.02, 0.02) 0.02 0.02	0.22 0.06 (0.17, 0.26) 0.17 0.34

PLS – periodontal ligament space width; EL—empty lacunae, ICI—inflammatory cell infiltrate, MT—mucosal thickness, C – coronal section, A – apical section, Ap – apical region, SD – standard deviation; CI – confidence interval

Twenty-one animals were included in the *Phase 1* analysis (De = 3, De + Be = 3, Zo = 5, Be = 3, Zo + Be = 2, Co = 5), and 34 animals were included in the Phase 2 analysis (De = 8, De + Be = 6, Zo = 2, Be = 7, Zo + Be = 4, Co = 7). In the Phase 1 analysis, 10 and 11 animals exhibited teeth in the upper and lower jaws, respectively, whereas the Phase 2 analysis considered only teeth in the upper jaw.

The estimated mean PLS-C values in the Co group amounted to 0.21 ± 0.11 mm and 0.15 ± 0.04 mm after Phase 1 and Phase 2, respectively. After Phase 1, the lowest PLS-C values were measured in the De group (0.14 ± 0.02 mm) and the highest in the Be group (0.31 ± 0.16 mm). After Phase 2, the mean PLS-C values ranged between 0.16 ± 0.02 mm (Zo group) and 0.23 ± 0.07 mm (Zo + Be). Linear regression analyses failed to show any significant differences among the groups at different time points (i.e., Phases 1 and 2). Time was also not found to be a predictor for the PLS-C measurements.

The PLS-A estimations in the Co group amounted to 0.15 ± 0.04 mm after Phase 1 and 0.14 ± 0.03 mm after Phase 2. The mean PLS-A values after phase 1 ranged between 0.14 ± 0.02 (De) and 0.24 ± 0.18 mm (Be). The corresponding values after Phase 2 varied between 0.12 ± 0.03 mm (Zo) and 0.19 ± 0.09 mm (Zo + Be). Linear regression analysis failed to show any significant differences across the groups at different time points. Time also did not have a significant influence on the assessed PLS-A values.

The EL-C values in the Co group were 8.8 ± 4.86 and 11.29 ± 40.6 after Phase 1 and Phase 2, respectively. The corresponding values in the test groups after Phase 1 ranged from 2.83 ± 0.76 (De group) to 6.5 ± 4.09 (Be group), and from 9.75 ± 7.94 (Be group) to 13.94 ± 4.48 (Zo + Be group) after Phase 2. Although the experimental group was not found to be associated with the EL-C measurements, time was found to be a significant factor influencing the EL-C values. In particular, the estimated EL-C measurements were two times higher after Phase 2 compared to those assessed after Phase 1 (*p* < 0.001). A similar tendency was noted for the EL-A and EL-Ap measurements, suggesting no significant difference between the Co and all test groups, but indicating significantly higher EL-A and EL-Ap values after Phase 2, in average by additional 5.94 (*p* < 0.001) and of 2.41 (*p* = 0.009) empty lacunae, respectively, compared to measurements assessed after Phase 1.

The presence of ICI was detected in 3 animals in the Co group (Phase 1: 1 animal; Phase 2: 2 animals) and in 18 animals in the test group (Phase 1: 4 animals; Phase 2: 14 animals). In terms of the ICI surface area, significant differences were noted between Zo, Be, and Zo + Be groups when compared to the Co. Superficially, the ICI surface area was 8.76 times higher in the Zo group (*p* = 0.016), 6.11 times higher in the Be group (*p* = 0.0015), and 53 times higher in the Zo + Be groups compared to that measured in the Co group. The time (i.e., Phases 1 and 2) was not found to be a predictor for the extent of the ICI area.

As for the MT measurements, the linear regression analysis showed a significant reduction of MT measurements by 23% (*p* = 0.002) after Phase 2 compared to those assessed after Phase 1. The differences among the experimental groups were not statistically significant.

When splitting the data by jaw, after Phase 1, comparable mean PLS, MT and EL values were measured at teeth in upper and lower jaws.

## Discussion

The present analysis aimed at investigating the influence of various antiresorptive and antiangiogenic medications on changes in periodontal and oral tissue structures. The histomorphometrical measurements of PLS, the number of EL, and the presence of ICI were measured at noninfected teeth in rats administered with various antiresorptive/antiangiogenic medications. The measurements were assessed after 17 (Phase 1) and 29 weeks (Phase) following the second systemic drug applications.

Based on the present findings, the width of PLS at both coronal and apical sections was comparable in all test and control groups after Phases 1 and 2. The present findings align with the outcomes of one previous experimental study in a mouse model, where unaltered periodontal spaces were detected in micro-CT scans of healthy molars of mice administered with either Za or RANKL inhibitors [20]. Opposite findings were observed at teeth with peri-radicular infections, suggesting the presence of significant alveolar bone loss and increased bone thickness in the presence of infection and drug administration (i.e. Za or RANKL inhibitors). The extraction of infectious teeth in animals receiving either Za or RANKL inhibitors was subsequently associated with persistent inflammatory infiltrates, bone exposure, and areas of osteonecrosis, whereas no adverse events were detected following the extraction of non-infectious teeth [20].

On the other hand, the findings of the present and aforementioned analysis contradict the results of a former volumetric study, which assessed the periodontal microstructure of noninfected teeth in rabbits administered with Za [14]. In particular, significantly wider PLS were measured in animals administered with Za compared to the control group (i.e., non-Za) [14]. The differences between Za and non-Za groups were more apparent for the PLS measurements in the upper jaw. Also, higher PLS values were measured in premolars as compared to molars. The discrepancies between the findings may be to some extent attributed to the different experimental models (i.e., rat and rabbit) and methodological approaches implemented for the measurements (i.e., micro-CT, 3D and histological), as the volumetric assessments may be more accurate compared to the 2-dimensional ones.

In the Co and all test groups, the EL measurements revealed a tendency toward a higher number of EL detected after Phase 2 as compared to Phase 1. An opposite tendency was noted for the MT assessments, which pointed toward significantly reduced MT measurements after Phase 2 as compared to those assessed after Phase 1. The latter results suggested that, over time, bone and mucosa undergo remodeling processes that were not influenced by the administration of the investigated medications. To the author’s best knowledge, this is the first study to investigate the effect of antiresorptive/antiangiogenic mediation on the EL and MT, which prevented any comparisons with previous findings.

Upon further analysis of the present data, ICI was considerably more frequently detected in the test groups compared to the Co group. After Phase 1, one animal in the Co group and four in the test group (Zo: 1, Be: 1, Zo + Be: 2) displayed the presence of ICI, which increased after Phase 2 to two animals in the Co group and 14 in the test group (De: 4, Be: 5, Zo + Be: 2, De + Be: 3). Furthermore, when assessing the surface area of ICI, significantly larger lesions were measured in the Zo + Be group, followed by the Zo and Be groups. Considering the present findings, it appears that the administration of bisphosphonates, antiangiogenic medication, and combinations thereof may be able to indicate inflammatory lesions in the absence of local inflammation related to the teeth. The present findings on the extent of ICI corroborate those assessed in the experimental study investigating the resolution of peri-implantitis lesions following the surgical treatment in the rodent model employing analogous experimental groups [18]. In particular, the largest ICIs could be detected in the Zo + Be group, followed by the De and De + Be groups [18]. In fact, as shown by the previous experimental studies, Zo is capable of inducting a proinflammatory signaling pathways and possesses proinflammatory effects [21, 22]. Clinically, infectious events have also been linked to the administration of Be, such as severe febrile neutropenia and fistulae/abscesses [23]. Accounting for the proinflammatory effects related to the administration of Be and Zo, the highest ICI values measured in the Bo, Be, and Zo + Be groups might at least be partially associated with the effect of the medications.

The major limitation of the present study is the lack of power calculations, which could not be performed due to the nature of the analysis. Furthermore, the absence of the subgroups of animals with infectious teeth did not allow to evaluate the role of local infections under the administration of antiresorptive/antiangiogenic mediations on changes in periodontal tissues. Additionally, the Phase 2 analysis only enrolled samples of maxillary teeth, because according to the original study protocol, only upper jaw samples were prepared for the histological processing. Thus, the potential differences in periodontal tissue structure with respect to the jaw (i.e., upper and lower) after a longer period of follow-up time could not be evaluated. Moreover, it should be noted that in the present analysis, antiresorptive/antiangiogenic medications were administered in healthy rats, without experimentally induced bone metabolic diseases, i.e., osteoporosis, which, in turn, may not reflect the clinical situation. Therefore, to mimic the clinical setting, future clinical studies may consider employing experimental models in animals with induced osteoporosis prior to the administration of mediations. Finally, when interpreting the present findings, particularly the PLS measurements, it is important to account for the distortions arising from the deviations in the angulation of the histological slices. Ideally, the slice of the histological specimens should follow the long axis of the tooth roots, which, however, cannot be standardized during the histological processing. Thus, in this perspective, the micro-CT (i.e., three-dimensional measurements) may provide considerably higher accuracy compared to the two-dimensional histological ones [24–26].

Within it’s limitations, the present evaluation was not able to find any morphological effects of different antiresorptive and antiangiogenic medications on periodontal and oral tissue structures. The presence of inflammatory cell infiltrates was more frequently observed in the animals administered with antiresorptive and antiangiogenic medications as well as combinations thereof.

the present evaluation was not able to find any morphological effects of different antiresorptive and antiangiogenic medications on periodontal and oral tissue structures.
